# Putative novel CSF biomarkers of Alzheimer’s disease based on the novel concept of generic protein misfolding and proteotoxicity: the PRAMA cohort

**DOI:** 10.1186/s40035-024-00405-0

**Published:** 2024-03-08

**Authors:** Alessandra Bigi, Giulia Fani, Valentina Bessi, Liliana Napolitano, Silvia Bagnoli, Assunta Ingannato, Lorenzo Neri, Roberta Cascella, Paolo Matteini, Sandro Sorbi, Benedetta Nacmias, Cristina Cecchi, Fabrizio Chiti

**Affiliations:** 1 Department of Experimental and Clinical Biomedical Sciences “Mario Serio”, Section of Biochemistry, University of Florence, Viale Morgagni 50, 50134 Florence, Italy; 2 Department of Neuroscience, Psychology, Drug Research and Child Health, University of Florence, Azienda Ospedaliero-Universitaria Careggi. Largo Brambilla, 3, 50134 Florence, Italy; 3 Institute of Applied Physics “Nello Carrara”, National Research Council, 50019 Sesto Fiorentino, Italy; 4 IRCCS Fondazione Don Carlo Gnocchi, Florence, Italy

Alzheimer’s disease (AD) accounts for 60%–70% of cases of dementia worldwide (https://www.alz.org). The NIA-AA (American National Institute of Aging and Alzheimer’s Association) has proposed a research framework based on a biomarker-grounded biological, rather than syndromal, definition of AD, where the disease has to be regarded as a continuum [1]. In this spectrum, seven biomarkers have attained widely recognized diagnostic relevance, including low levels of the 42-residue amyloid beta (Aβ_42_) and high concentrations of total tau (T-tau) and phosphorylated tau (P-tau) in the cerebrospinal fluid (CSF), high cortical amyloid deposition and tau deposition measured with positron emission tomography (PET), poor brain glucose metabolism measured with fluoro-deoxyglucose PET, and significant brain atrophy imaged with magnetic resonance imaging. Based on these biomarkers, the AT(N) system (A for Aβ deposition, T for pathologic tau, and N for neurodegeneration) has been proposed for biological characterization and staging of AD [1].

Research and identification of novel biomarkers are important to enrich the aforementioned research framework, but also as diagnostic tools for supporting the existing biomarkers that often produce uncertain diagnoses in early AD. They are also important to address the pathological complexity and heterogeneity of the disease, and to enrich our biomarker list with others with more prognostic value [2].

All the classical protein-based biomarkers reveal the soluble and aggregation states of specific proteins, such as Aβ_42_, T-tau and P-tau. However, it is recognized that protein misfolding diseases, including AD, are characterized by a generic failure of the proteostasis network (PN), which physiologically maintains proteins in a soluble non-aggregated state [3–5]. In a compromised PN status, a great number of proteins lose solubility and gain a propensity to misfold and aggregate [4–6]. Accumulation of protein aggregates is both an effect and a cause of PN decline, driving a vicious cycle that ultimately leads to its collapse [3, 4, 6]. Consistently, in every neurodegenerative disease the main characterizing protein deposits are often associated with those of other proteins.

Building on this idea, in this work we compared CSF samples extracted from AD and non-AD cases in a novel Italian study named PRAMA (Proteomics, RAdiomics & Machine learning-integrated strategy for precision medicine for Alzheimer’s). We sought the presence of aggregated protein species, detectable with biophysical methods, and proteotoxicity, in the form of misfolded protein oligomers able to cause cell dysfunction to cultured cells using cell viability assays, to identify novel biomarkers of AD. This idea was based on the detection of misfolded proteins not just of the Aβ_42_ and tau proteins that represent a very small fraction of the protein population composing the CSF, but of the overall CSF proteome.

Twenty-nine patients with final diagnosis of AD with evidence of AD pathophysiological processes and 20 patients with final diagnosis of other diseases affecting the central nervous system were recruited. CSF samples were collected and treated (Additional file 1: Materials and methods). Patient diagnosis was uncertain at the time of CSF collection and was ascertained only after clinical-neuropsychological examination and CSF biomarker support. None of the final diagnoses were post-mortem. The mean and individual demographic characteristics of both groups, values of the classical CSF biomarkers (levels of P-tau, T-tau, Aβ_42_ and Aβ_42_/Aβ_40_ ratio), percentages of patients with the ε4 allele of the Apolipoprotein E (*APOE*) gene and scores of mini-mental state examination (MMSE) tests are shown in Additional file 1: Tables S1 and S2.

The total protein concentration in the CSF, measured with the Bradford assay, ranged from approximately 0.2 to 1.0 mg/ml in both groups, consistent with previous analyses [7]. The mean values were 0.46 ± 0.22 and 0.44 ± 0.19, respectively, indicating similar distributions in the two groups. Scatter plots of Aβ_42_/Aβ_40_ *versus* T-tau and Aβ_42_/Aβ_40_ *versus* P-tau, with the thresholds (t*) derived from optimization of the Youden’s indexes of the two parameters (horizontal and vertical lines, respectively), showed a good separation between non-AD and AD cases, with the latter having higher T-tau and P-tau and lower Aβ_42_/Aβ_40_, as expected (Additional file 1: Fig. S1a, b,* P* < 0.0001, Fisher’s exact test [FET] and Chi-square test [CST]). The areas under the curve (AUCs) in the receiver operating characteristic (ROC) curves were 0.858, 0.885 and 0.882 for Aβ_42_/Aβ_40_, T-tau and P-tau, respectively (Fig. S1c). This analysis validates our cohort as it indicates that the two groups are good representatives of non-AD and AD cases, respectively.

CSFs were first compared by measuring the size distributions of their particles with dynamic light scattering (DLS), as shown here for five representative non-AD and five representative AD patients (Fig. 1a). In both groups, a peak of small species having an apparent hydrodynamic diameter (*D*_*h*_) of ~10 nm was evident, which arose from the dominant largest CSF proteins, such as human serum albumin. However, large species arising from protein aggregates were also present in both groups, all having *D*_*h*_ values around or higher than 100 nm. The light scattering intensity (*LSI*) arising from large species (*D*_*h*_ > 30 nm) was generally higher in AD cases, indicating a larger proportion of protein aggregates in this group (Fig. 1a). When considering all non-AD and AD cases, the large species accounted for 60% ± 19% and 74% ± 20% of* LSI*, respectively (Fig. 1b), and the difference was highly significant (*P* = 0.01, Mann–Whitney test [MWT]).

**Fig. 1:**
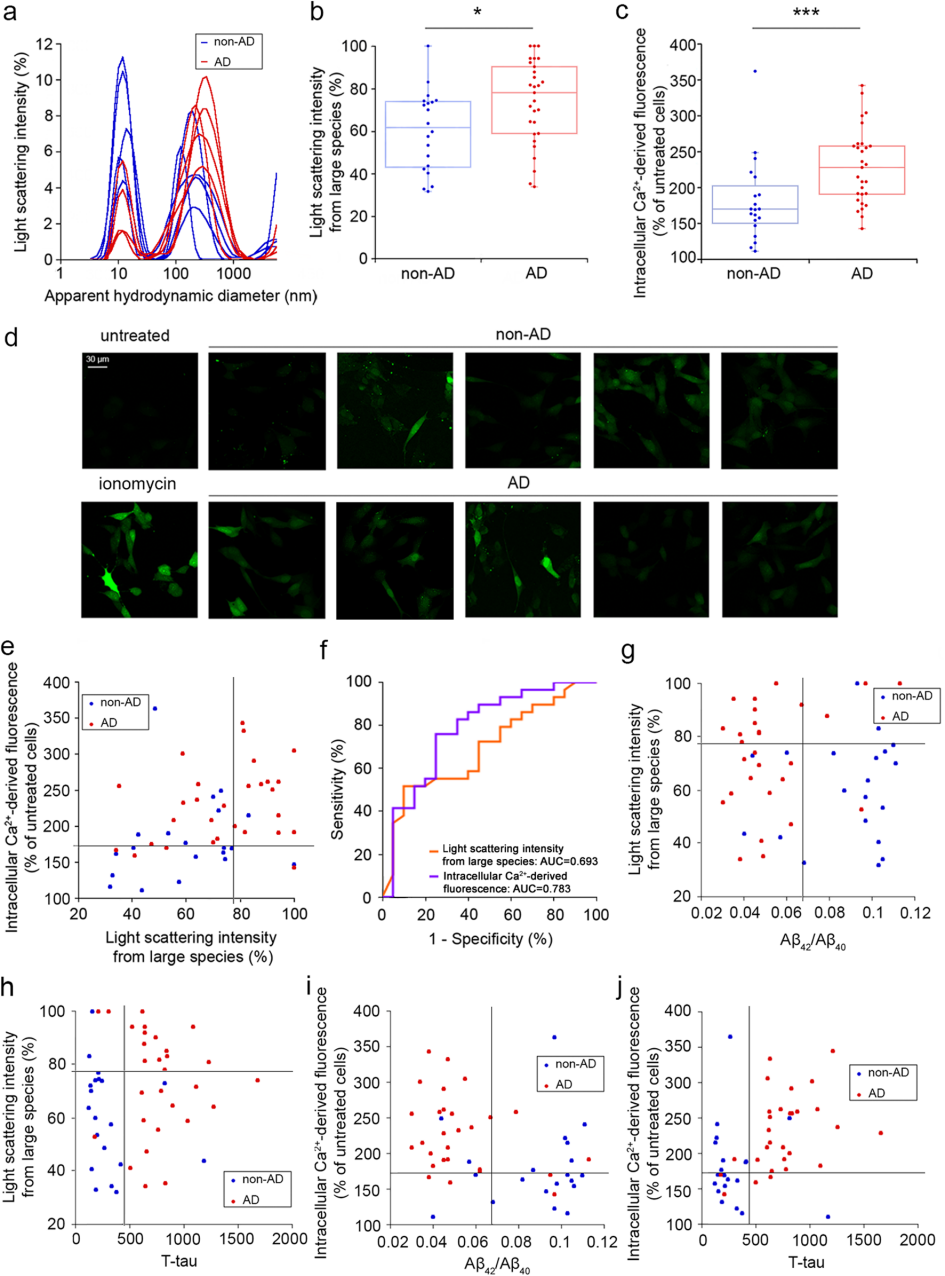
**a** DLS-detected size distributions of particles in CSF samples from five representative non-AD (blue) and five representative AD patients (red). **b** Box plots reporting the DLS *LSI* values derived from large species in all non-AD and AD CSFs (*P < 0.05, MWT). **c** Box plots reporting the Ca^2+^-derived fluorescence values in all non-AD and AD CSFs (***P < 0.001, MWT). **d** Confocal microscopic images showing the intracellular Ca^2+^ levels (green) in SH-SY5Y cells treated with the same CSFs as in **a**. Untreated cells and cells treated with ionomycin are negative and positive controls, respectively. **e** Scatter plots for intracellular Ca^2+^ levels *versus* *LSI* from large protein species. Horizontal and vertical lines indicate the thresholds t* derived from optimization of the Youden’s indexes of the two shown parameters. **f** ROC curves of the two parameters with AUC values. **g–j** Scatter plots of the indicated classical and novel biomarkers. Information as in **e**

Since protein aggregates added to the extracellular medium of cultured cells have the ability to bind and destabilize biological membranes and cause an influx of Ca^2+^ ions into the cytosol [8, 9], the levels of intracellular Ca^2+^ ions in cultured cells exposed to CSF samples are a good indicator of CSF proteotoxicity [9]. We therefore added the CSF samples to the culture medium of SH-SY5Y neuroblastoma cells (*v*:*v* 1:1) and measured the intracellular Ca^2+^ levels after 5 h using the Fluo-4 AM probe and confocal fluorescence microscopy (Fig. 1d). The Ca^2+^ levels ranged from approximately 110% to 250% in cells treated with non-AD CSFs (except one outlier sample) and from approximately 140% to 340% in those treated with AD CSFs, relative to untreated cells (Fig. 1c). The mean values in the two groups were 181% ± 57% and 229% ± 53%, respectively, with very highly significant difference (*P* = 0.0003, MWT). We also screened other potential biophysical and biological biomarkers, based on circular dichroism spectroscopy, intrinsic tryptophan fluorescence and MTT-reduction assay for cell viability, but no significant difference was found between the two groups (Additional file 1: Fig. S2).

Hence, the AD CSFs were characterized by higher values of *LSI* from large protein species in the DLS distributions and higher ability to induce high cytosolic Ca^2+^ levels when added to the medium of cultured cells. These findings can be explained with the presence of higher amounts of large protein particles and misfolded protein oligomers inducing Ca^2+^ dyshomeostasis in cells, respectively. The scatter plot of intracellular Ca^2+^ levels *versus* *LSI* from large protein species, with the thresholds (t*) derived from optimization of the Youden’s indexes of the two parameters as horizontal and vertical lines, respectively, indicated a good separation between non-AD and AD cases (Fig. 1e). The three quadrants above one or both t* values contained a disproportionate amount of AD *versus* non-AD cases, whereas the quadrant below both t* values contained mainly non-AD cases, with few AD CSFs (*P* = 0.001 with FET; *P* = 0.0003 with CST). Consequently, a diagnosis of AD based on pairs of these CSF parameters had a high sensitivity and a medium specificity. The AUC values under the ROC curves were 0.693 and 0.783 (> 0.5) for *LSI* and Ca^2+^, respectively (Fig. 1f).

We then combined the three classical CSF biomarkers (Aβ_42_/Aβ_40_, T-tau, P-tau) with the two novel putative biomarkers identified here (*LSI*, Ca^2+^ levels). Scatter plots for all possible pairs are shown in Fig. 1g–j and Fig. S3a,b. The combination of *LSI* or Ca^2+^ levels with the three classical biomarkers effectively distinguished between AD and non-AD populations. In plots involving *LSI*, the best level of diagnosis, in terms of both sensitivity and specificity, was achieved when considering non-AD cases only in one quadrant. This quadrant was the bottom-right for *LSI* *versus* Aβ_42_/Aβ_40_ (Fig. 1g) and the bottom-left for *LSI* *versus* T-tau or P-tau (Fig. 1h, Additional file 1: Fig. S3a). By contrast, in plots involving Ca^2+^ levels, the best outcome was achieved when considering AD cases only in the top-left quadrant for Ca^2+^ *versus* Aβ_42_/Aβ_40_ (Fig. 1i) and the top-right for Ca^2+^ *versus* T-tau or P-tau (Fig. 1j and Additional file 1: Fig. S3b). Separations were very highly significant in all cases (*P* < 0.001 with both FET and CST).

In conclusion, these results extend our attention from individual specific proteins to the status of the entire proteome in the CSF for the assessment of an AD-associated biological profile. We identified large protein species in the CSF (detectable with DLS) and toxic oligomers (detectable as an increase of Ca^2+^ influx in cultured cells) as two novel AD biomarkers. The AD/non-AD segregation using these two novel biomarkers is very highly significant (*P* ≤ 0.001). In the broader context of AD, these results also reinforce the view that the PN is compromised in AD. PN failure not only leads to an aggregated and proteotoxic status of Aβ and tau, but also to many other proteins of the entire proteome. Longitudinal evaluation of PN alteration along with the classical biomarkers will be important for elucidating the cause-effect relationship between PN failure and Aβ/tau misfolding and assessing how early the PN dysfunction is in the context of the disease.

These two novel biomarkers will be applied to a larger PRAMA cohort and possibly other cohorts in the attempt to optimize our parameters, as well as to evaluate how they can be combined with traditional biomarkers to gain sensitivity and specificity for AD diagnosis. It will also be important to assess whether these two novel biomarkers are extendable to plasma samples, and usable as prognostic tools. Finally, it will be important to assess whether they are exploitable for early AD diagnosis in preclinical and even preceding phases, on the grounds that a defective PN is considered to be an early event in protein misfolding diseases and even a cause of the formation of large aggregates such as amyloid plaques and neurofibrillary tangles.

### Supplementary Information

**Additional file 1**. Materials and methods. **Table S1.** Mean genetic, demographic and clinical characteristics and biomarker levels of the non-AD and AD patients. **Table S2.** Individual genetic, demographic and clinical characteristics and biomarker levels of the non-AD and AD patients. **Fig. S1** Scatter plots for Aβ_42_/Aβ_40_ ratio *versus* T-tau and P-tau. **Fig. S2** Box plots reporting the wavelength of maximum intrinsic fluorescence (*λ*_*max*_), the ellipticity at 222 nm (θ_222_) for the CSF samples, and the MTT reduction values in SH-SY5Y cells treated for 24 h with CSF samples from all non-AD and all AD patients. **Fig. S3** Scatter plots for *LSI* from large species and intracellular Ca^2+^-derived fluorescence *versus* P-tau.

## Data Availability

All data generated or analysed during this study are included in this published article and its supplementary information file.

## Abbreviations

Aβ: Amyloid-beta
AD: Alzheimer’s disease
CSF: Cerebrospinal fluid
CST: Chi-square test
DLS: Dynamic light scattering
FET: Fisher’s exact test
LSI: Light scattering intensity
MMSE: Mini-Mental State Examination
MWT: Mann-Whitney test
PET: Position emission tomography
PN: Proteostasis network
ROC: Receiver operating characteristic
T-tau: Total tau
