# Supplementary material for: Putative novel CSF biomarkers of Alzheimer’s disease based on the novel concept of generic protein misfolding and proteotoxicity: the PRAMA cohort
Source: Transl Neurodegener. 2024 Mar 8;13:14. doi: 10.1186/s40035-024-00405-0 (PMC10924410; doi:10.1186/s40035-024-00405-0)
Supplement: Supplementary file 1 — Additional file 1. Materials and methods. Table S1. Mean genetic, demographic and clinical characteristics and biomarker levels of the non-AD and AD patients. Table S2. Individual genetic, demographic and clinical characteristics and biomarker levels of the non-AD and AD patients. Fig. S1 Scatter plots for Aβ42/Aβ40 ratio versus T-tau and P-tau. Fig. S2 Box plots reporting the wavelength of maximum intrinsic fluorescence (λmax), the ellipticity at 222 nm (θ222) for the CSF samples, and the MTT reduction values in SH-SY5Y cells treated for 24 h with CSF samples from all non-AD and all AD patients. Fig. S3 Scatter plots for LSI from large species and intracellular Ca2+-derived fluorescence versus P-tau. [file 40035_2024_405_MOESM1_ESM.docx]

**Supplementary Material**

**Materials and methods**

**Patients**

Between October 2020 and May 2023, we collected 52 CSF samples from patients referred to the Centre for Alzheimer’s Disease and Adult Cognitive Disorders of Careggi Hospital in Florence. Patients met the following inclusion criteria: 29 patients who received a final diagnosis of AD dementia according to the NIA-AA criteria (McKhann et al., 2011) or a diagnosis of MCI according to NIA-AA criteria (Albert et al., 2011). 20 patients with final diagnosis of other diseases affecting the central nervous system (CNS) but without having a clear involvement of protein misfolding or aggregation (non-AD cases) have participated to this study: 19 with idiopathic normal pressure hydrocephalus (iNPH) according to international guidelines (Relkin et al., 2005), 1 patient with multiple sclerosis (MS) and 1 with vascular dementia (VD), all diagnosed according to international guidelines (Thompson et al., 2018; Neary et al., 1998; McKeith et al., 2017; Sachdev et al., 2014). None of the diagnoses were *post-mortem*.

**Neuropsychological examination**

Neuropsychological examination included global measurements of Mini-Mental State Examination (MMSE), Digit and Visuo-spatial Span forward, Rey Auditory Verbal Learning Test, Trail Making Test A, attentional matrices, language and phonemic fluency task, constructional praxis, such as Rey-Osterrieth complex figure copy and Clock test, and executive function tests, such as Trail Making test B, Stroop Test, and Frontal Assessment Battery (Giacomucci et al., 2022).

**CSF collection and biomarkers analysis**

CSF samples were collected by lumbar puncture, then immediately centrifuged at 200 g for 1 min, 20 °C, then at 4000 g for 10 min at 4 °C, and the supernatant was further used for novel biomarkers analyses. The pellet contained generally tiny amounts of proteins. An aliquot of the samples was stored at − 80 °C until analysis for classical biomarkers. Aβ_42_ levels, Aβ_42_/Aβ_40_ ratio and T-tau and P-tau levels were measured using a chemiluminescent enzyme immunoassay (CLEIA) analyzer LUMIPULSE G600: Lumipulse G β-Amyloid 1–40, Lumipulse G β-Amyloid 1–42, Lumipulse G Total Tau, and Lumipulse G P-Tau 181, with all reagent kits obtained from Fujirebio. At the time of CSF collection and analysis for novel biomarkers, diagnosis was uncertain and was ascertained only later, following neuropsychological and clinical examination with classical CSF- and imaging-based biomarkers. Separation between AD and non-AD cases was carried out only after the end of all analyses. In some cases, the initial diagnostic suspect was not confirmed and such cases were transferred to the other group.

**Apolipoprotein E ε4 genotyping**

A standard automated method (QIAcube, QIAGEN) was used to isolate DNA from peripheral blood samples. *APOE* genotypes were investigated by high-resolution melting analysis (HRMA) (Sorbi et al., 1994). Two sets of PCR primers were designed to amplify the regions encompassing rs7412 [NC_000019.9:g[M13] [GG14] 0.45412079C > T] and rs429358 (NC_000019.9:g.45411941 T > C). The samples with known *APOE* genotypes, which had been validated by DNA sequencing, were used as standard references.

**Bradford assay**

Protein concentration was determined by the Bradford assay (Bradford et al., 1976). In brief, 50 µl solutions containing increasing concentrations of bovine serum albumin (BSA, Sigma-Aldrich), ranging from 0 to 2 mg/ml, were used to develop a standard curve; 1:5 diluted Protein Assay Dye Reagent (Bio-Rad) was added to these solutions and the measured absorbance at 595 nm was then plotted *versus* the corresponding BSA concentration. The resulting equation was used to calculate CSF protein concentration after measuring the absorbance at 595 nm of 3 μl of CSF supernatant diluted in 47 μl of H_2_O in the presence of the Protein Assay Dye Reagent.

**DLS measurements**

Size distribution profiles of CSFs were recorded at 25 °C using a Zetasizer Nano S dynamic light scattering (DLS) device (Malvern Panalytical) thermostated with a Peltier temperature controller and using a 10 mm reduced-volume plastic cell (50 µL volume). The refractive index and viscosity were 1.45 and 0.82 cp, respectively. These values were determined from the Malvern analysis software after entering the typical CSF composition previously reported (Robertson, 2010). The measurements were acquired with cell position 3.00 and attenuator index 10. All DLS size distributions (*LSI* *versus* *D_h_*) were the means of three acquisitions (*n=*3) and were analysed both automatically, with a cut-off value at 30 nm to identify large species, but also by visual inspection one by one to remove anomalous distributions among the three acquired for each sample, to identify the first large peak attributable to the pool of soluble species, therefore better estimating the *LSI* from large species in each mean distribution, so that a more precise quantification of the light scattering intensity arising from large species could be measured in each CSF sample.

**Measurement of intracellular Ca^2+^**

Authenticated human SH-SY5Y neuroblastoma cells were purchased from A.T.C.C. and cultured in Dulbecco’s modified Eagle’s medium (DMEM), F-12 Ham with 25 mM 4-(2-Hydroxyethyl) piperazine-1-ethanesulfonic acid (HEPES) and NaHCO_3_ (1:1) supplemented with 10% fetal bovine serum (FBS), 1 mM glutamine and 1% antibiotics, as reported previously (Capitini et al. 2014; Bigi et al., 2020). Cells were maintained in a 5% CO_2_ humidified atmosphere at 37 °C and grown until 80% confluence for a maximum of 20 passages. Cells were plated on 24-well plates containing glass coverslips (40.000 cells per well). After 24 h, they were washed with PBS and then treated for 5 h with 75 μl of CSFs mixed 1:1 with 75 µl of cell culture medium (150 µl final volume), or for 2 h with 1 μM ionomycin in a culture medium (75 µl) mixed 1:1 with PBS (75 µl), or with culture medium (75 µl) mixed 1:1 with PBS (75 µl) without additives (referred to as “untreated”). Cells were then washed with PBS and loaded with 4.5 μM Fluo-4 AM (Thermo Fisher Scientific) for 10 min. Following incubation, the cytosolic Ca^2+^ levels were detected in 4-6 images for each sample (*n=*4-6) after excitation at 488 nm by a TCS SP8 scanning confocal microscopy system (Leica Microsystems) equipped with an argon laser source. All data were normalized to untreated cells (taken as 100%), whereas cells treated with ionomycin (measured here as 677%) provide useful references for re-normalization in other labs. A series of 1-μM-thick optical sections (1024 × 1024 pixels) was taken through the cell depth for each sample using a Leica Plan Apo 63× oil immersion objective, and all sections were projected as a single composite image by superimposition. Pinhole diameters, detector gain and laser powers were optimized. Images were analyzed with ImageJ (NIH) software (Rasband 1997–2018). Fluorescence intensities were expressed as the percentage of that measured in untreated cells.

**Intrinsic Tryptophan Fluorescence Assay**

Intrinsic tryptophan fluorescence spectra of CSFs were acquired at 25 °C from 300 to 500 nm after excitation at 280 nm (slits of 5 and 5 nm, respectively), using a 0.3 × 0.3 cm black wall quartz cell (50 µl volume) on an Agilent Cary Eclipse spectrofluorometer (Agilent Technologies) equipped with a thermostated cell holder attached to an Agilent PCB 1500 water Peltier system.

**Far-UV circular dichroism**

CSF samples were analyzed at 25 °C using a Jasco J-810 Spectropolarimeter equipped with a thermostated cell holder attached to a Thermo Haake C25P water bath and using a 0.1 cm path-length cell. Far-UV circular dichroism spectra and the high tension (HT) spectra were collected over the 200-260 nm wavelength range. It was not possible to record the spectra at wavelength values lower than 200 nm, because of the high absorbance values (HT > 700 V) recorded at these wavelength values. All spectra were blank subtracted and plotted as non-normalised ellipticity (θ) values (mdeg units) *versus* wavelength (nm). Spectra were not normalised to mean residue ellipticity ([θ]_res_) because CSF samples are mixtures of proteins, each with a well-defined molecular weight.

**MTT reduction assay**

SH-SY5Y cells were seeded on 96-well plates at a density of 10.000 cells per well and the 3-(4,5-dimethylthiazol-2-yl)-2,5-diphenyl-2H-tetrazolium bromide (MTT) reduction was evaluated as reported previously (Capitini et al. 2014; Bigi et al., 2023). Briefly, cells were treated for 24 h with 50 µl of CSFs mixed 1:1 with 50 µl of cell culture medium (100 µl final volume), or with culture medium (50 µl) mixed 1:1 with PBS (50 µl) without additives (referred to as “untreated”). Cells were then washed with PBS and the MTT solution was added to the culture medium for 4 h. After this time, a cell lysis buffer (20% sodium dodecyl sulfate and 50% N,N-dimethylformamide, pH 4.7) was added to each well to solubilize formazan crystalline precipitates; 1 h later, the absorbance values of blue formazan were measured at 595 nm by using an enzyme-linked immunosorbent assay plate reader (Bio-Rad). MTT reduction values were expressed as the percentage of those measured in untreated cells.

**Receiver operating characteristic curve and area under the curve analysis**

The values of two given parameters X and Y were plotted in a scatter plot for both non-AD and AD CSFs. For each of the two parameters the Youden’s index *J* was determined as:

*J* = sensitivity + specificity - 1 = *q(t)* + 1 - *p(t)* - 1 = *q(t)* + *p(t)* (1)

where *t* is a given mobile threshold, *q(t)* is the “sensitivity” and corresponds to the probability of a true positive, *1- p(t)* is the “specificity” and correspond to the probability of a true negative. Equation 1 corresponds to:

*J* = [*n*_true positives_ / (*n*_true positives_ + *n*_false negatives_)] + [*n*_true negatives_ / (*n*_true negatives_ + *n*_false positives_)] – 1 (2)

where *n*_true positives_ is the number of true positives and so on. The optimal threshold *t** of a given parameter in a test is provided by moving *t* so that J is maximum:

*J* = max [*q(t)* + *p(t)*] (3)

t

The ROC curve was edited by plotting *J* for a moving *t* value in a graph of *q(t)* (sensitivity) *versus* *p(t)* (1-specificity), and the AUC was calculated using the GraphPad Prism 5.0 software.

**Statistical analysis**

Values of each parameter in non-AD and AD groups were expressed as means ± standard deviation (S.D.). A one-tailed Mann Whitney *t* test (MWT) was performed to compare non-AD *versus* AD values for each parameter (GraphPad Prism 10.0 software). *P* < 0.05, 0.01, 0.001 were considered to be statistically significant (*), highly statistically significant (**) and very highly statistically significant (***), respectively. Populations of non-AD and AD CSFs were categorised in two groups each, on the grounds of whether their individual values for a given parameter were below or above the threshold value (*t**) or occupied a given quadrant in scatter plots. This produced a total of four groups for each analysis, two for AD and two for non-AD CSFs. The significance of the separation between non-AD and AD CSFs was evaluated using the one-tailed Fisher’s exact test and the Chi-square test (GraphPad Prism 10.0 software). *P* < 0.05, 0.01, 0.001 were considered as above.

**Supplementary References**

Albert MS, DeKosky ST, Dickson D, Dubois B, Feldman HH, Fox NC et al. The diagnosis of mild cognitive impairment due to Alzheimer's disease: recommendations from the National Institute on Aging-Alzheimer's Association workgroups on diagnostic guidelines for Alzheimer's disease. Alzheimers Dement. 2011;7:270-279.

Bigi A, Loffredo G, Cascella R, and Cecchi C. Targeting pathological amyloid aggregates with conformation sensitive antibodies. Curr Alzheimer Res. 2020;17:722–34.

Bigi A, Cascella R, Fani G, Bernacchioni C, Cencetti F, Bruni P et al. Sphingosine 1-phosphate attenuates neuronal dysfunction induced by amyloid-β oligomers through endocytic internalization of NMDA receptors. FEBS J. 2023;290:112-133.

Bradford MM. A rapid and sensitive method for the quantitation of microgram quantities of protein utilizing the principle of protein-dye binding. Anal Biochem. 1976;72:248-54.

Capitini C, Conti S, Perni M, Guidi F, Cascella R, De Poli A et al. TDP-43 inclusion bodies formed in bacteria are structurally amorphous, non-amyloid and inherently toxic to neuroblastoma cells. PLoS One. 2014;9:e86720.

Giacomucci G, Galdo G, Polito C, Berti V, Padiglioni S, Mazzeo S et al. Unravelling neural correlates of empathy deficits in Subjective Cognitive Decline, Mild Cognitive Impairment and Alzheimer's Disease. Behav Brain Res. 2022;428:113893.

McKhann GM, Knopman DS, Chertkow H, Hyman BT, Jack CR Jr, Kawas CH et al. The diagnosis of dementia due to Alzheimer's disease: recommendations from the National Institute on Aging-Alzheimer's Association workgroups on diagnostic guidelines for Alzheimer's disease. Alzheimers Dement. 2011;7:263-9.

McKeith IG, Boeve BF, Dickson DW, Halliday G, Taylor JP, Weintraub D et al. Diagnosis and management of dementia with Lewy bodies: fourth consensus report of the DLB Consortium. Neurology. 2017;89:88–100.

Neary D, Snowden JS, Gustafson L, Passant U, Stuss D, Black S et al. Frontotemporal lobar degeneration: a consensus on clinical diagnostic criteria. Neurology. 1998;51:1546–1554.

Relkin N, Marmarou A, Klinge P, Bergsneider M, and Black PM. Diagnosing Idiopathic Normal-Pressure Hydrocephalus. Neurosurgery. 2005;57:S4-16.

Robertson DS. The physical chemistry of brain and neural cell membranes: An overview. Neurochem Res. 2010;35:681–687.

Sachdev P, Kalaria R, O'Brien J, Skoog I, Alladi S, Black SE et al. Diagnostic criteria for vascular cognitive disorders: a VASCOG statement. Alzheimer Dis Assoc Disord. 2014;28:206-18.

Thompson AJ, Banwell BL, Barkhof F, Carroll WM, Coetzee T, Comi G et al. Diagnosis of multiple sclerosis: 2017 revisions of the McDonald criteria. Lancet Neurol. 2018;17:162-173.

**Supplementary Tables**

**Table S1.** Mean (± standard deviations where indicated) genetic, demographic and clinical characteristics and biomarker levels of the non-AD and AD patients.

|  | **ApoE ε4+ ^a^** | **Age at CSF collection (years)** | **P-tau**  **(pg/ml)** | **T-tau**  **(pg/ml)** | **Aβ_1-42_**  **(pg/ml)** | **Aβ_1-42_**  **/Aβ_1-40_** | **MMSE score ^b^** |
| --- | --- | --- | --- | --- | --- | --- | --- |
| **Non-AD** | 15.8% (*n=*19) | 72.6 ± 7.0 (*n=*18) | 42.4 ± 43.2 (*n=*20) | 300.0 ± 261.3 (*n=*20) | 647.5 ± 313.5 (*n=*20) | 0.089 ± 0.022 (*n=*20) | 28.7 ± 2.1  (*n=*3) |
| **AD** | 40.7% (*n=*27) | 70.2 ± 7.3 (*n=*29) | 126.9 ± 60.6 (*n=*29) | 763.1 ± 315.0 (*n=*29) | 675.0 ± 295.9 (*n=*29) | 0.053 ± 0.020 (*n=*29) | 19.7 ± 5.7  (*n=*25) |
| ***P* value ^c^** |  | *P* > 0.05 | *P*<0.0001 | *P*<0.0001 | *P*>0.05 | *P*<0.0001 | *P*=0.0009 |

^a^ All patients underwent *APOE* genotyping: *APOE* genotype was coded as *APOE* ε4- (no *APOE* ε4 alleles) and *APOE* ε4+ (presence of one or two *APOE* ε4 alleles). The column reports the percentage of *APOE* ε4+ genotype and the number of patients analysed (n).

^b^ The MMSE score ranges for any patient from 0 to 30, with 30 indicating high brain performance. The *n* value of non-AD cases is low because there was no clinical indication to perform a MMSE test in many cases.

^c^ determined with one-tailed Mann-Withney test (MWT)

**Table S2.** Individual genetic, demographic and clinical characteristics and biomarker levels of the non-AD and AD patients.

|  | **Patient ID** | **Clinical diagnosis** | ***APOE***  **genotype ^a^** | **Age at CSF collection (years)** | **P-tau**  **(pg/ml)** | **T-tau**  **(pg/ml)** | **Aβ_1-42_**  **(pg/ml)** | **Aβ_1-42_**  **/Aβ_1-40_** | **MMSE score ^b^** |
| --- | --- | --- | --- | --- | --- | --- | --- | --- | --- |
| **Non-AD** | 877 | VD | 3/3 | 75 | 45.6 | 377 | 1288 | 0.103 | 30.0 |
|  | 879 | HyC | 3/3 | 79 | 42.2 | 328 | 1243 | 0.097 | 26.3 |
|  | 892 | HyC | 2/3 | 76 | 27.8 | 183 | 305 | 0.060 | 30.0 |
|  | 896 | HyC | 3/4 | 85 | 168.8 | 1184 | 723 | 0.040 |  |
|  | 932 | MS | 3/3 | 65 | 31.9 | 268 | 1006 | 0.097 |  |
|  | 933 | HyC | 2/3 | 80 | 154.3 | 820 | 702 | 0.044 |  |
|  | 937 | HyC | 3/3 | 80 | 24.4 | 186 | 501 | 0.087 |  |
|  | 947 | HyC | 3/3 | 73 | 27 | 203 | 723 | 0.105 |  |
|  | 963 | HyC | 2/3 | 75 | 17.2 | 215 | 491 | 0.106 |  |
|  | 971 | HyC | 3/4 | 59 | 15.7 | 151 | 336 | 0.103 |  |
|  | 1011 | HyC | 3/4 | 68 | 33.4 | 190 | 349 | 0.068 |  |
|  | 1016 | HyC | 3/3 | 71 | 15.4 | 123 | 388 | 0.098 |  |
|  | 1019 | HyC | 3/3 | 71 | 40.7 | 336 | 1238 | 0.105 |  |
|  | 1023 | HyC | 2/3 | 71 | 35.4 | 245 | 747 | 0.082 |  |
|  | 1033 | HyC | 3/3 | 59 | 21.4 | 210 | 612 | 0.110 |  |
|  | 1039 | HyC | 3/3 | 73 | 17.6 | 126 | 456 | 0.103 |  |
|  | 1046 | HyC | 3/3 | 78 | 21.1 | 156 | 480 | 0.093 |  |
|  | 1078 | HyC | 3/3 | 68 | 15.4 | 142 | 390 | 0.111 |  |
|  | 1079 | HyC | 3/3 |  | 17.2 | 139 | 473 | 0.102 |  |
|  | 1088 | HyC |  |  | 75 | 418 | 499 | 0.057 |  |
| **AD** | 834 | AD | 3/3 | 69 | 117.3 | 759 | 391 | 0.030 | 26.0 |
|  | 835 | AD | 3/3 | 81 | 86.6 | 524 | 677 | 0.045 | 15.5 |
|  | 836 | AD | 3/4 | 80 | 110.7 | 640 | 470 | 0.035 | 23.4 |
|  | 837 | AD | 4/4 | 65 | 96.6 | 619 | 592 | 0.055 | 18.9 |
|  | 853 | AD | 4/4 | 77 | 98.3 | 646 | 265 | 0.038 | 16.3 |
|  | 857 | AD | 3/4 | 76 | 85 | 639 | 592 | 0.067 | 11.3 |
|  | 861 | AD | 3/3 | 74 | 324.2 | 1679 | 800 | 0.045 | 23.7 |
|  | 867 | AD | 4/4 | 62 | 175.9 | 1083 | 584 | 0.045 |  |
|  | 870 | AD | 3/3 | 74 | 111.7 | 657 | 726 | 0.062 | 23.7 |
|  | 873 | AD | 3/3 | 82 | 180 | 1031 | 566 | 0.034 | 18.5 |
|  | 883 | AD | 4/4 | 63 | 153.7 | 829 | 588 | 0.049 |  |
|  | 887 | AD | 2/3 | 72 | 27.8 | 211 | 1024 | 0.097 | 25.7 |
|  | 890 | AD | 3/3 | 78 | 84.6 | 634 | 1498 | 0.079 | 15.0 |
|  | 897 | AD | 3/3 | 66 | 134.6 | 821 | 347 | 0.039 | 19.2 |
|  | 900 | AD | 3/4 | 71 | 102 | 641 | 525 | 0.047 | 21.3 |
|  | 910 | MCI | 3/3 | 68 | 18.3 | 178 | 825 | 0.095 |  |
|  | 919 | AD | 3/3 | 72 | 143 | 892 | 685 | 0.043 | 19.7 |
|  | 925 | AD | 3/4 | 69 | 78.7 | 507 | 573 | 0.048 | 16.9 |
|  | 928 | AD | 3/4 | 75 | 134 | 840 | 663 | 0.045 | 23.7 |
|  | 931 | AD | 3/4 | 67 | 175.4 | 1228 | 748 | 0.038 | 19.2 |
|  | 961 | AD | 3/3 | 73 | 214.8 | 1273 | 876 | 0.058 | 11.3 |
|  | 1020 | MCI | 2/3 | 55 | 41 | 305 | 1598 | 0.113 | 23.9 |
|  | 1036 | MCI | 3/4 | 72 | 109.7 | 627 | 667 | 0.052 | 26.3 |
|  | 1037 | MCI | 3/3 | 75 | 136.8 | 738 | 788 | 0.045 | 27.7 |
|  | 1047 | AD | 3/3 | 57 | 206.8 | 1114 | 504 | 0.040 | 23.0 |
|  | 1056 | AD | 3/3 | 77 | 141.6 | 787 | 653 | 0.062 | 4.3 |
|  | 1060 | AD |  | 57 | 103.8 | 613 | 372 | 0.047 |  |
|  | 1085 | AD | 2/3 | 60 | 148.7 | 844 | 352 | 0.03 | 23.8 |
|  | 1086 | AD |  | 69 | 137.4 | 772 | 627 | 0.047 | 13.7 |

^a^ All patients underwent *APOE* genotyping: 2,3,4 indicated the alleles ε2, ε3 and ε4, respectively.

^b^ The MMSE score ranges for any patient from 0 to 30, with 30 indicating high brain performance. The *n* value of non-AD cases is low because there was no clinical indication to perform a MMSE test in many cases.

**Supplementary figure legends**


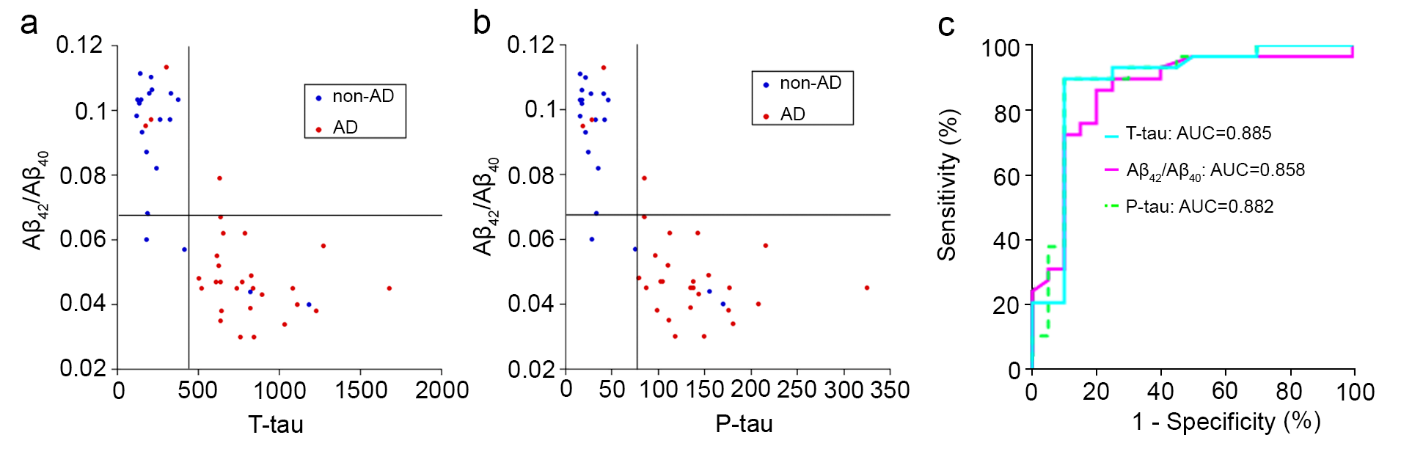


**Fig. S1** Scatter plots for Aβ_42_/Aβ_40_ ratio *versus* T-tau (**a**) and P-tau (**b**). (**c**) ROC curves of Aβ_42_/Aβ_40_ ratio, T-tau and P-tau. AUC values are indicated for each parameter. AD CSFs (red) and non-AD CSFs (blue) categorized to occupy the bottom-right quadrant and the remaining three quadrants, respectively, provide a good separation between non-AD and AD patients.


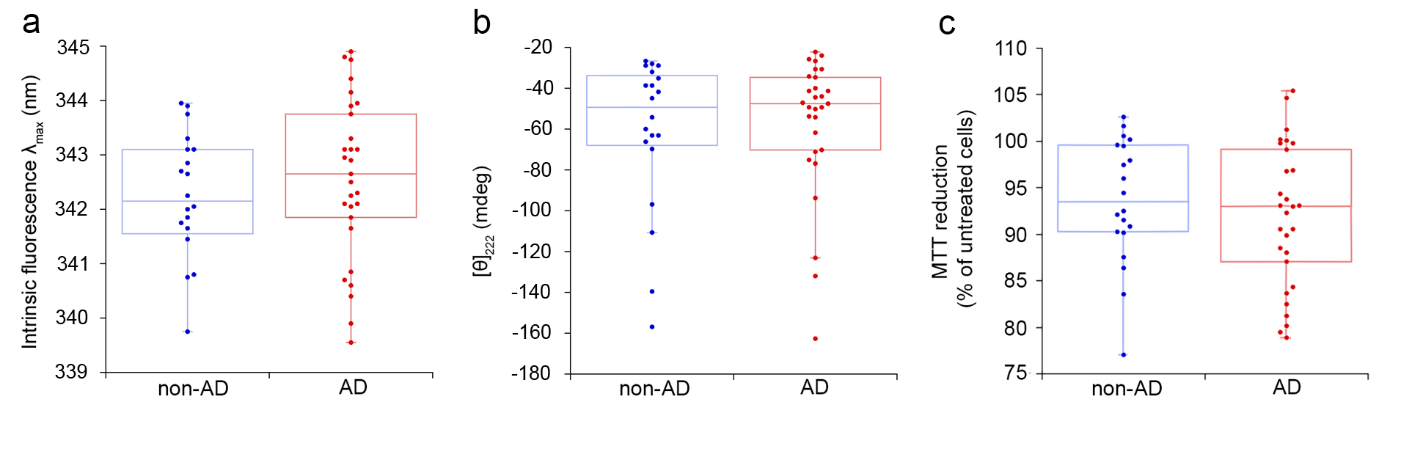


**Fig. S2** Box plots reporting the wavelength of maximum intrinsic fluorescence (*λ_max_*) (**a**), the ellipticity at 222 nm (*θ_222_*) measured for the CSF samples (**b**), and the MTT reduction values in SH-SY5Y cells treated for 24 h with CSF samples (**c**) from all non-AD and all AD patients (**P*>0.05 in all three cases, MWT). **a** The *λ_max_* values ranged from 340 to 344 nm in non-AD cases and from 340 to 345 nm in AD cases. The mean values in the two groups were 342.2±1.2 nm and 342.6±1.5 nm, respectively, indicating the absence of a significant difference (*P*=0.32, MWT). **b** The *θ_222_* values ranged from *ca*. -22 to *ca*. -162 mdeg (cell 0.1 cm) in both groups. Their mean values were -59±36 and -57±33 in non-AD and AD cases, respectively, indicating the absence of a significant difference (*P* =0.42, MWT).**c** Both non-AD and AD cases caused small decreases of MTT reduction relative to untreated cells, with values from *ca*. 77% to *ca*. 103% and from *ca*. 79% to *ca*. 105% in the two groups, respectively. Thus, they were found to behave similarly with average values of 93.5±6.3% and 92.0±7.7%, respectively, indicating the absence of a significant difference (*P*=0.25, MWT).


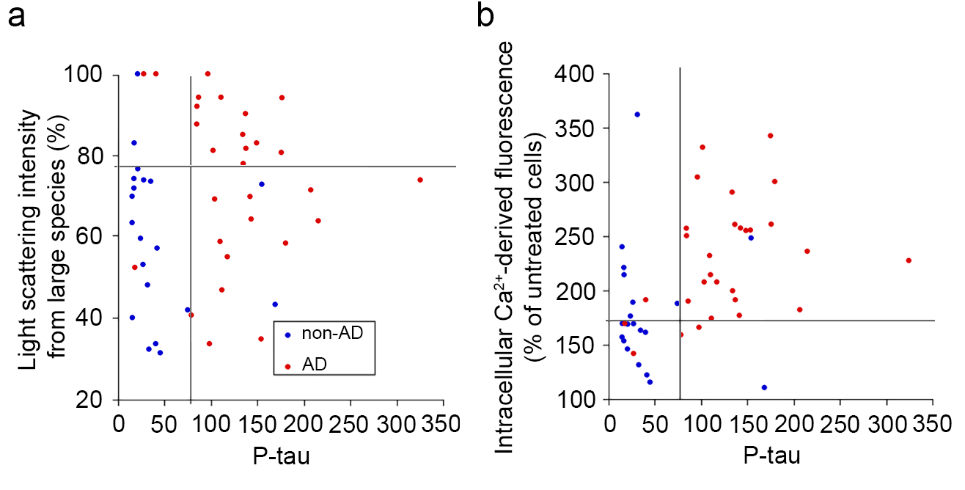


**Fig. S3** Scatter plots for *LSI* from large species (**a**) and intracellular Ca^2+^-derived fluorescence (**b**) *versus* P-tau. In both cases, the optimized thresholds (*t**) derived from optimization of the Youden’s indexes of each couple of parameters are represented as horizontal and vertical lines, respectively. In panel **a**, the non-AD (blue) and AD (red) CSF samples were categorized to occupy the bottom-left quadrant and the remaining three quadrants, respectively, providing a good separation between non-AD and AD patients. In panel **b**, the AD (red) and non-AD (blue) CSF samples were categorized to occupy the top-right quadrant and the remaining three quadrants, respectively, providing a good separation between AD and non-AD patients.
